# Supplementary material for: Effect of Oral Administration of Polyethylene Glycol 400 on Gut Microbiota Composition and Diet-Induced Obesity in Mice
Source: Microorganisms. 2023 Jul 26;11(8):1882. doi: 10.3390/microorganisms11081882 (PMC10456793; doi:10.3390/microorganisms11081882)
Supplement: Supplementary file 1 [file microorganisms-11-01882-s001.zip › microorganisms-2493089-supplementary.pdf]

**Table S1. Primer sequences for qPCR**

| Target gene or bacteria | Direction | Sequence (5′ to 3′) |
|---|---|---|
| Rpl13a | Forward | CCATTGTGGCCAAGCAGGTA |
| | Reverse | CTCGGGAGGGGTTGGTATTC |
| TNF-a | Forward | TAGCCCACGTCGTAGCAAAC |
| | Reverse | GCAGCCTTGTCCCTTGAAGA |
| Adiponectin | Forward | GTCTGGCTCCAGGTGTATGG |
| | Reverse | AGCTGAAAGTGTGTCGACTGT |
| F4/80 | Forward | CTGGGATCCTACAGCTGCTC |
| | Reverse | AGGAGCCTGGTACATTGGTG |
| CD11c | Forward | CTGGATAGCCTTTCTTCTGCTG |
| | Reverse | GCACACTGTGTCCGAACTCA |
| Tjp1 | Forward | GCCAGAGAAAAGTTGGCAAG |
| | Reverse | TTGGATACCACTGCGCATAA |
| Muc2 | Forward | GCTGACGAGTGGTTGGTGAATG |
| | Reverse | GATGAGGTGGCAGACAGGAGAC |
| Eubacterium | Forward | ACTCCTACGGGAGGCAGCAGT |
| | Reverse | ATTACCGCGGCTGCTGGC |
| Akkermansia muciniphila | Forward | CAGCACGTGAAGGTGGGGAC |
| | Reverse | CCTTGCGGTTGGCTTCAGAT |
| Parabacteroides goldsteinii | Forward | GCAGCACGATGTAGCAATACA |
| | Reverse | TTAACAAATATTTCCATGTGGAAC |

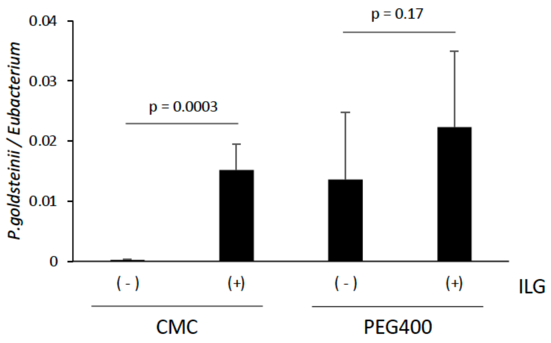


**Figure S1. Relative abundance of *P. goldsteinii* in mice administered with ILG dispersed in CMC or PEG400.** RT-PCR analysis of eubacterium and *P. goldsteinii* in fecal DNA isolated from ND-fed mice administered with CMC or PEG400 (40%) in the presence or absence of ILG ($n$ = 6). ILG dispersed in CMC or PEG400 was administered for two weeks post-acclimation.
